# Early reduction in PD-L1 expression predicts faster treatment response in human cutaneous leishmaniasis

**DOI:** 10.1172/JCI142765

**Published:** 2021-10-05

**Authors:** Nidhi S. Dey, Sujai Senaratne, Vijani Somaratne, Nayani P. Madarasinghe, Bimalka Seneviratne, Sarah Forrester, Marcela Montes de Oca, Luiza Campos Reis, Srija Moulik, Pegine B. Walrad, Mitali Chatterjee, Hiro Goto, Renu Wickremasinghe, Dimitris Lagos, Paul M. Kaye, Shalindra Ranasinghe

**Affiliations:** 1 York Biomedical Research Institute, Hull York Medical School, University of York, York, United Kingdom.; 2 Department of Parasitology, University of Sri Jayewardenepura, Nugegoda, Sri Lanka.; 3 Dermatology Unit, District General Hospital Embilipitiya, Embilipitiya, Sri Lanka.; 4 Dermatology Unit, Teaching Hospital Anuradhapura, Anuradhapura, Sri Lanka.; 5 Deparment of Pathology, University of Sri Jayewardenepura, Nugegoda, Sri Lanka.; 6 Instituto de Medicina Tropical de São Paulo, Faculdade de Medicina, Universidade de Sao Paulo, Sao Paulo, Brazil.; 7 Department of Pharmacology, Institute of Postgraduate Medical Education and Research, Kolkata, India.; 8 York Biomedical Research Institute, Department of Biology, University of York, York, United Kingdom.; 9 Departamento de Medicina Preventiva, Faculdade de Medicina, Universidade de São Paulo, São Paulo, Brazil.

**Keywords:** Immunology, Infectious disease, Immunotherapy, Molecular pathology, Parasitology

## Abstract

Cutaneous leishmaniasis (CL) is caused by *Leishmania donovani* in Sri Lanka. Pentavalent antimonials (e.g., sodium stibogluconate [SSG]) remain first-line drugs for CL with no new effective treatments emerging. We studied whole blood and lesion transcriptomes from Sri Lankan patients with CL at presentation and during SSG treatment. From lesions but not whole blood, we identified differential expression of immune-related genes, including immune checkpoint molecules, after onset of treatment. Using spatial profiling and RNA-FISH, we confirmed reduced expression of programmed death-ligand 1 (PD-L1) and indoleamine 2,3-dioxygenase 1 (IDO1) proteins on treatment in lesions of a second validation cohort and further demonstrated significantly higher expression of these checkpoint molecules on parasite-infected compared with noninfected lesional CD68^+^ monocytes and macrophages. Crucially, early reduction in PD-L1 but not IDO1 expression was predictive of rate of clinical cure (HR = 4.88) and occurred in parallel with reduction in parasite load. Our data support a model whereby the initial anti–leishmanial activity of antimonial drugs alleviates checkpoint inhibition on T cells, facilitating immune-drug synergism and clinical cure. Our findings demonstrate that PD-L1 expression can be used as a predictor of rapidity of clinical response to SSG treatment in Sri Lanka and support further evaluation of PD-L1 as a host-directed therapeutic in leishmaniasis.

## Graphical abstract

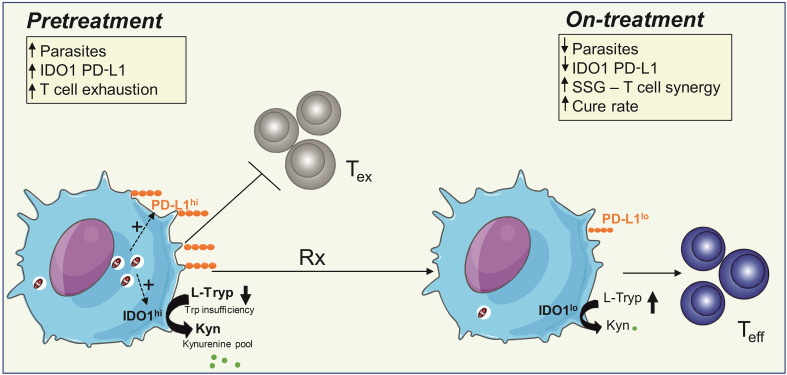

## Introduction

Every year, approximately 600,000 to 1 million new cases of cutaneous leishmaniasis (CL) occur, with a broad global distribution, often leading to stigma and reduced life chances and placing a burden on health services (1–3). Treatment options for CL have changed little in the more than 70 years since pentavalent antimonial drugs were first introduced, and there are limited new treatments on the horizon (3). Sri Lanka is endemic for CL, with the first autochthonous case being reported in 1992 (4). Sri Lankan CL is caused by *Leishmania donovani* zymodeme MON-37 parasite (5–7), usually associated with visceral leishmaniasis in other endemic countries. Current treatment for CL in Sri Lanka involves weekly intralesional or daily intramuscular administration of sodium stibogluconate (SSG), with or without cryotherapy, based on the site and size of the lesion and response to treatment. Cure often takes many months, and some patients may fail to respond completely or withdraw from treatment (8).

Most of our understanding of the host immune response in CL stems from experimental models, and human disease is much less understood (9). Immune checkpoint molecules have been implicated in disease progression in preclinical models (10–17), but their role in human CL has not been explored. It is widely proposed that immune-drug synergy is required for effective treatment and that host-directed therapy (HDT) may have a future role in patient management (18–20), but few validated targets have emerged. Here, we searched for early correlates of treatment response that might be used to stratify patient response. Our results indicate an intimate relationship between intracellular parasitism and immune checkpoint molecule expression, with programmed death-ligand 1 (PD-L1) emerging as a promising target for HDT in Sri Lanka.

## Results and Discussion

**Figure 1:**
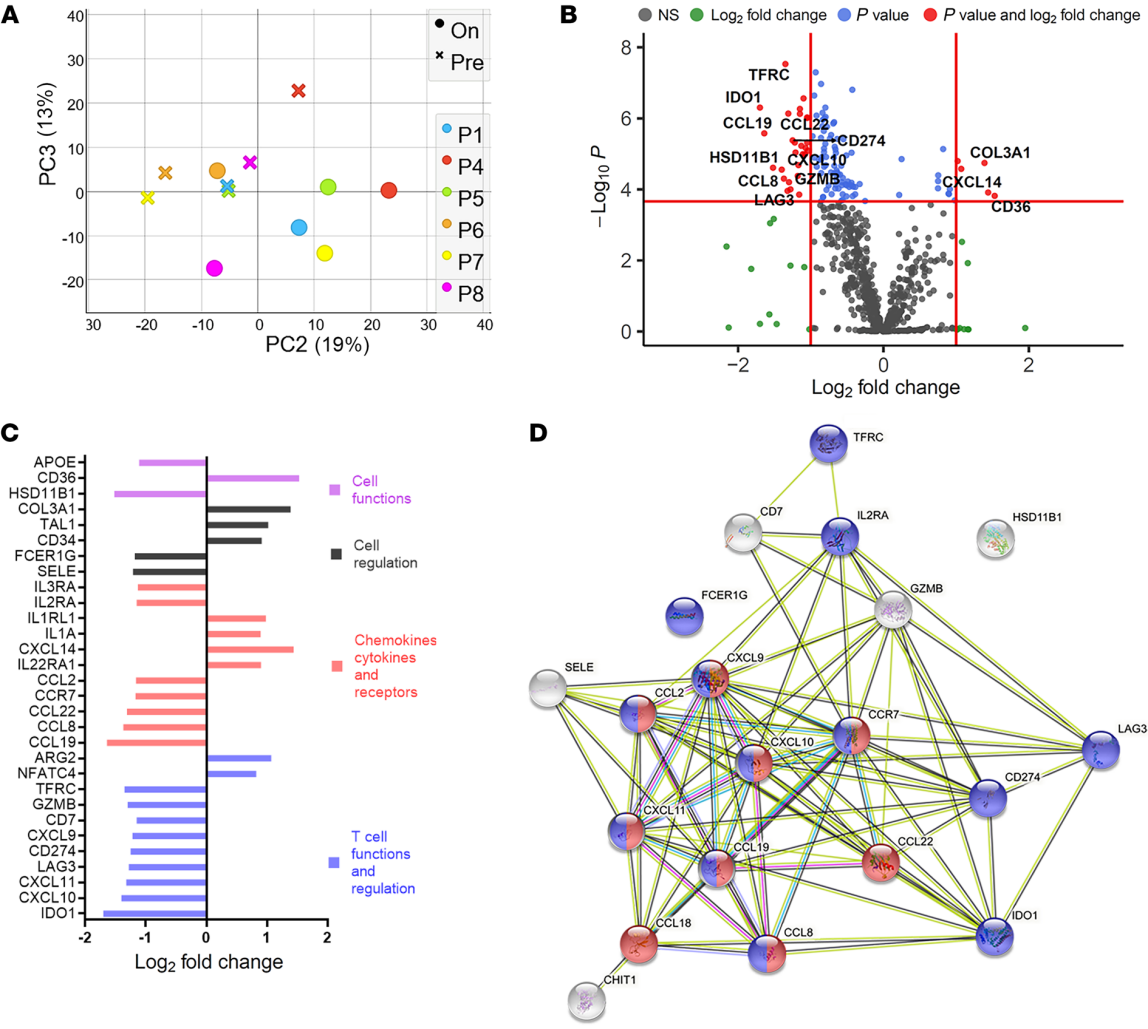
Differential expression and network analysis of genes regulated by drug treatment in lesions of Sri Lankan patients with CL. Immune-targeted tissue transcriptomics were conducted on tissue sections from test cohort patients comparing transcriptomes at presentation and on treatment. (**A**) Principal component analysis was performed to show differences between the pre- and on-treatment transcriptome of each patient based on 770 genes from the nCounter PanCancer Immunology Panel (*n =* 6). (**B**) DEGs comparing pretreatment biopsies with biopsies taken after 2 weeks on treatment (SSG). Cut off (red line) drawn at equivalent of adjusted *P* = 0.01 and log (fold change) of 1. (**C**) Top 30 genes that changed in expression on SSG treatment. (**D**) STRING protein-protein interaction network (https://string-db.org; ref. 22) analysis of genes listed in Supplemental Table 3 downregulated on SSG treatment. Pathways represent GO: 0072676, lymphocyte migration (red spheres), and GO: 0002684, positive regulation of immune system process (blue spheres). Top 20 genes are shown (log_2_ fold change ≥ 1.15) for clarity.

We first conducted a targeted transcriptomic analysis of the lesion site in a test cohort of 6 patients with typical homogeneous nodulo-ulcerative CL lesions (3 females, 3 males; mean age ± SD, 34 ± 11 years; (Supplemental Figures 1–3 and Supplemental Table 1; supplemental material available online with this article; https://doi.org/10.1172/JCI142765DS1). Principal component analyses of lesion transcriptomic data showed separation of pre- and on-treatment samples in most patients (Figure 1A) and 120 differentially expressed genes (DEGs) were identified (FDR adjusted *P* < 0.01; Figure 1B). In contrast, no DEGs were identified by RNA-seq in whole blood (Supplemental Figure 4 and Supplemental Table 2), suggesting that unlike CL caused by *L*. *braziliensis* (21), CL due to *L*. *donovani* in Sri Lanka is not accompanied by an overt systemic immune response.

Following treatment, the majority of DEGs in dermal lesions were downregulated (87%; 105/120), suggesting a reduction in inflammation following treatment (105 downregulated, 15 upregulated; Figure 1B and Supplemental Table 3). Genes for cellular functions and regulation, chemokines, membrane receptors, and T cell function and regulation were among the top 20 DEGs (Figure 1C). Further, STRING analysis (22) identified lymphocyte migration (Gene Ontology database [GO]: 0002687, FDR = 1.06 × 10^–14^; including interferon inducible chemokines such as *CXCL9*, *CXCL10*, *CXCL11*, *CCL19*, *CCL8*) and regulators of immune response (GO: 0002684, FDR = 1.94 × 10^–11^; including *IDO1*, *LAG3,* and *CD274/PDL1*) as highly enriched pathways (Figure 1D). Transcripts of inflammatory mediators including *CXCL10*, *GZMB*, *CCL2*, and *CCR7* (receptor for CCL19), previously shown to be associated with other forms of murine (23–25) or human (26–28) CL were also downregulated with initiation of treatment (Supplemental Table 3).

**Figure 2:**
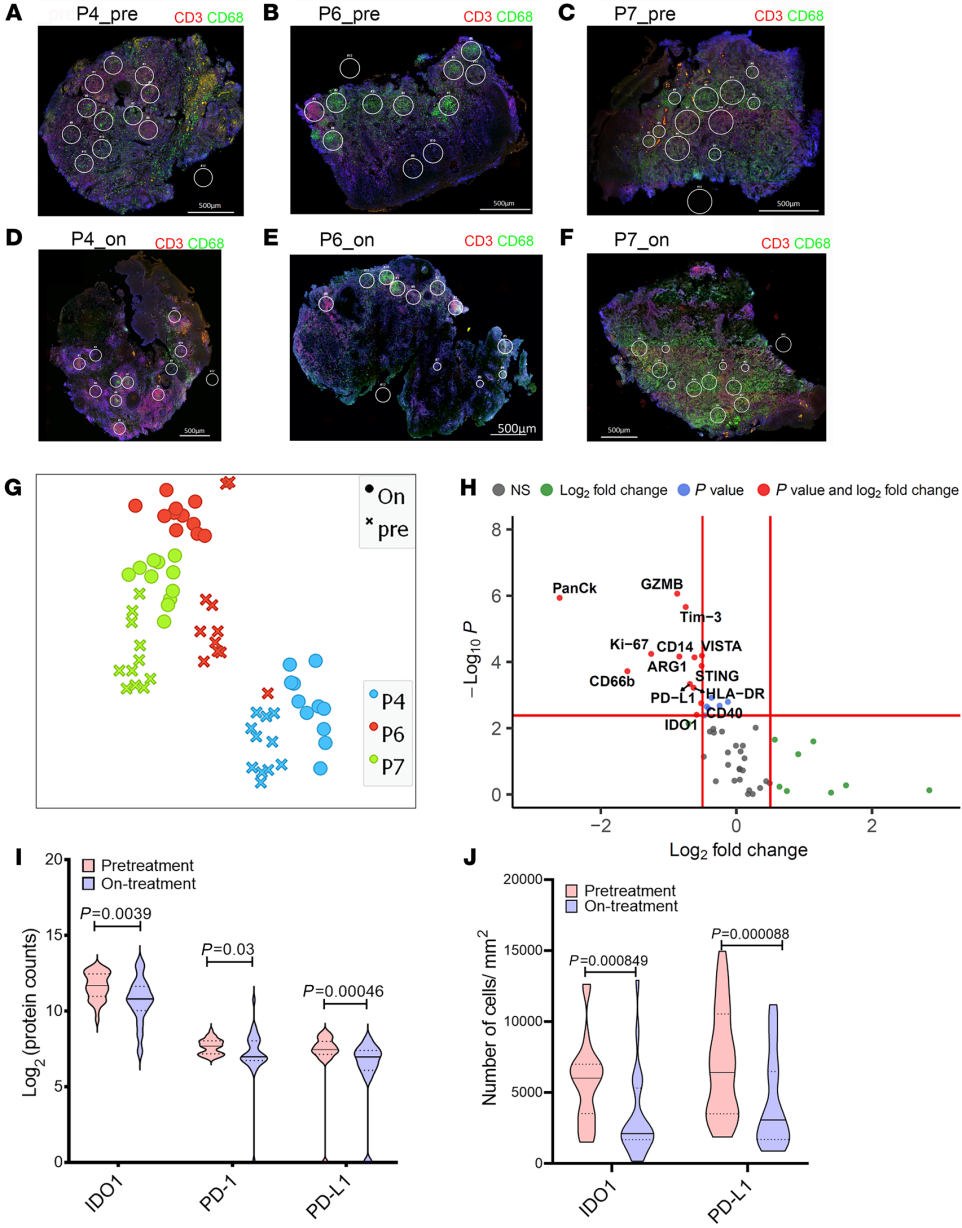
Digital spatial profiling of CL lesions. Digital spatial profiling was performed on tissue sections from test cohort individuals comparing ROIs from pre and on-treatment biopsies. (**A**–**F**) ROIs on CD3^+^ and/or CD68^+^ rich areas from pre- and on-treatment biopsies from patients P4, P6, and P7 (CD68, green; CD3, red; Syto13, blue). Original magnification: ×20; scale bar: 500 μm. (**G**) t-SNE plot based on 20 PCA loadings colored on patient ID. (**H**) Differential protein expression analysis comparing pretreatment to on-treatment ROIs. Red lines indicate adjusted *P* value cut off of 1% (Mann-Whitney test with FDR correction based on Benjamini, Krieger, and Yekutieli 2-stage set-up method) and log_2_ fold change = 0.5 (*n =* 33 ROIs). (**I**) IDO1, PD-1, and PD-L1 expression in pre- and on-treatment ROIs. Mann Whitney rank test (*n =* 33 ROIs). (**J**) Immunohistochemistry (IHC) was performed on sections from patients in the validation cohort (*n =* 23) before and during treatment, and quantitated using StrataQuest (see Methods). Comparisons were by Wilcoxon matched-pairs signed rank test. Dotted lines show upper and lower quantile in **I** and **J**, median by solid line.

We next conducted multiplexed antibody digital spatial profiling (29) for 59 immune targets, selecting regions of interest (ROIs) based on expression of CD3^+^ and/or CD68^+^ (Supplemental Figure 5 and Figure 2, A–F). The t-SNE dimensional reduction on a total of 33 ROIs analyzed from 3 patients (P4, P6, and P7; Figure 2G) indicated a considerable degree of interpatient heterogeneity in pretreatment lesional protein profiles, but with clear discrimination for each patient between pre- and on-treatment ROIs. Upon treatment, indoleamine 2,3-dioxygenase 1 (IDO1) and PD-L1 as well as PD-1 were selectively reduced in expression (Figure 2, H and I). STRING analysis of all discoveries based on FDR (5%) also indicated significant enrichment in GO: 002684, as well as a pathway associated with regulation of T cell activation (GO: 0050863; Supplemental Figure 6, A and B).

As IDO1 and PD-L1 have been targeted in cancer immunotherapy and hold promise for drug repurposing, we next sought to further validate these findings using quantitative IHC in an independent cohort of patients with CL (5 females, 18 males; mean age ± SD, 44 ± 11 years; time to diagnosis, 7.76 ± 8.2 months; Supplemental Figures 7 and 8 and Supplemental Table 4) sampled at baseline and after 4 weeks of treatment. Using an accepted cut-off of greater than 5% of cells being positive (30), all patients (*n =* 23) expressed IDO1 (histochemical score [H-score] median = 81.2; range 16–165; ref. 31) and 20 of 23 patients had a reduction in the abundance of IDO1^+^ cells on treatment (H-score median = 32; range 1–171; *P =* 0.0023; Figure 2J). All patients were PD-L1 positive at presentation (*n =* 23; H-score median = 82.8; range 12–164) and 20 of 23 patients exhibited a reduction in the number of PD-L1–expressing cells on treatment (Figure 2J; H-score median = 36.7; range 12.3–36.7; *P =* 0.0008). Collectively, these data indicate that IDO1 and PD-L1 are highly expressed in the lesions of Sri Lankan patients with CL and reduction in expression of these 2 checkpoint molecules represents an early response to SSG.

**Figure 3:**
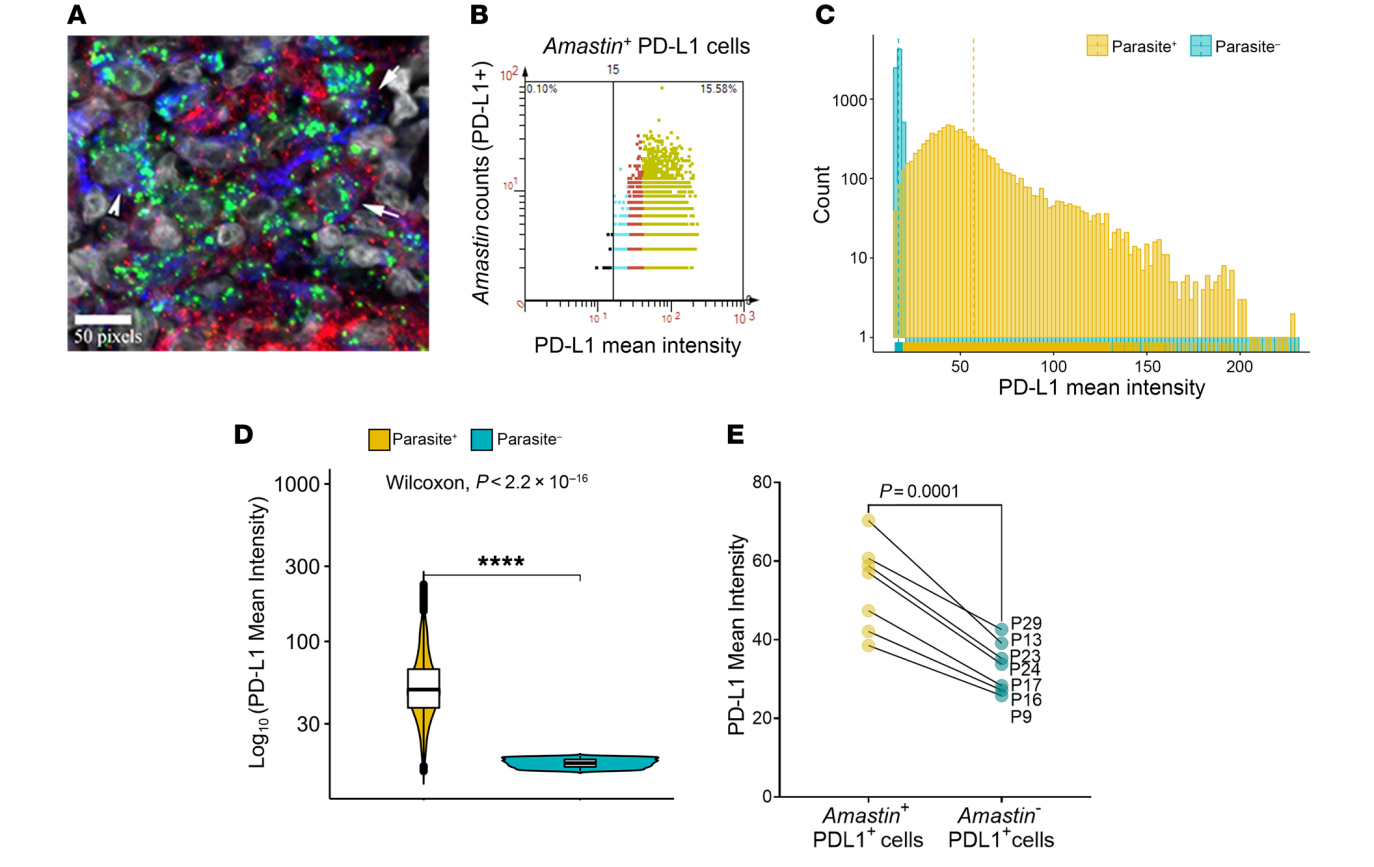
Immunofluorescence analyses of PD-L1 in infected and uninfected cells. Dual IHC-FISH using an *Amastin* probe was performed on pretreatment sections of patients enrolled in the validation cohort. (**A**) A 400× confocal image showing infection of PD-L1^+^CD68^+^ (arrows) and PD-L1^–^CD68^+^ (arrowhead) cells. Scale bar: 50 pixels. (**B**) Relationship between PD-L1 expression and parasite burden (*Amastin* dot count). Scattergram from a representative patient (P24 at presentation) showing *Amastin*^+^ low (cyan), medium (red), and high (green) PD-L1–expressing cells with respect to parasite abundance. (**C**) Fluorescence intensity distributions of infected and uninfected PD-L1 cells. (**D**) Mean fluorescence intensity of PD-L1 expression on *Amastin*^–^ cells compared with *Amastin*^+^ cells from representative patient P24. The upper and lower whisker represents highest and lowest value that is within 1.5 times the interquartile range. *n =* 9159 parasite positive cells and *n =* 41520 parasite negative cells. Significance score was generated using Wilcoxon signed rank test. (**E**) PD-L1 expression on *Amastin*^+^PD-L1^+^ cells versus *Amastin^–^*PD-L1^+^ cells (*n =* 7 patients). Significance score was generated using Student’s 2-tailed paired *t* test after testing for normality using Shapiro-Wilk and Kolkogorov-Smirnov tests.

Though in vitro studies have indicated that intracellular parasitism by *Leishmania* could impact on the expression of immune checkpoint molecules (32–34), this has not been established in situ during human disease. To address this question, we combined IHC with RNA-FISH (35) to identify *Amastin* transcripts (as a surrogate for viable amastigotes) with a bespoke StrataQuest image analysis pipeline (Supplemental Figure 9, A–F). In 7 patients studied who were *Amastin*^+^ at presentation (Supplemental Methods), PD-L1 expression colocalized with CD68^+^ macrophages and parasitized cells were both PD-L1^+^ and PD-L1^–^ (Figure 3A). We binned the Amastin^+^ PDL1^+^ and Amastin^–^ PDL1^+^ cells based on PD-L1 mean fluorescence intensity (Figure 3, B–D) and found that cells containing abundant *Amastin* transcripts expressed more PD-L1 than cells with fewer or no *Amastin* transcripts (Figure 3, B–E, Supplemental Figure 9, G–L, and Supplemental Figure 10). To independently corroborate this observation, we showed that a Sri Lankan strain of *L*. *donovani* was also capable of inducing upregulation of PD-L1 expression on human monocyte–derived macrophages in vitro (Supplemental Figure 11, A–F), as previously described for *L*. *major* (34). Similarly, IDO1 extensively colocalized with CD68^+^ cells (Supplemental Figure 12A) and both IDO1^+^CD68^+^ and IDO1^–^CD68^+^ cells were infected (Supplemental Figure 12B). Using a similar gating strategy (Supplemental Figure 12, C–H; *n =* 3 patients), we found that cells with abundant *Amastin* transcripts expressed more IDO1 than those with fewer or no *Amastin* transcripts (Supplemental Figure 12, I–K). These data show that, although a notable population of uninfected CD68^+^ cells contribute to PD-L1 and IDO1 expression within CL lesions, intracellular parasitism leads to heightened expression of these checkpoint molecules in lesional monocytes and macrophages.

**Figure 4:**
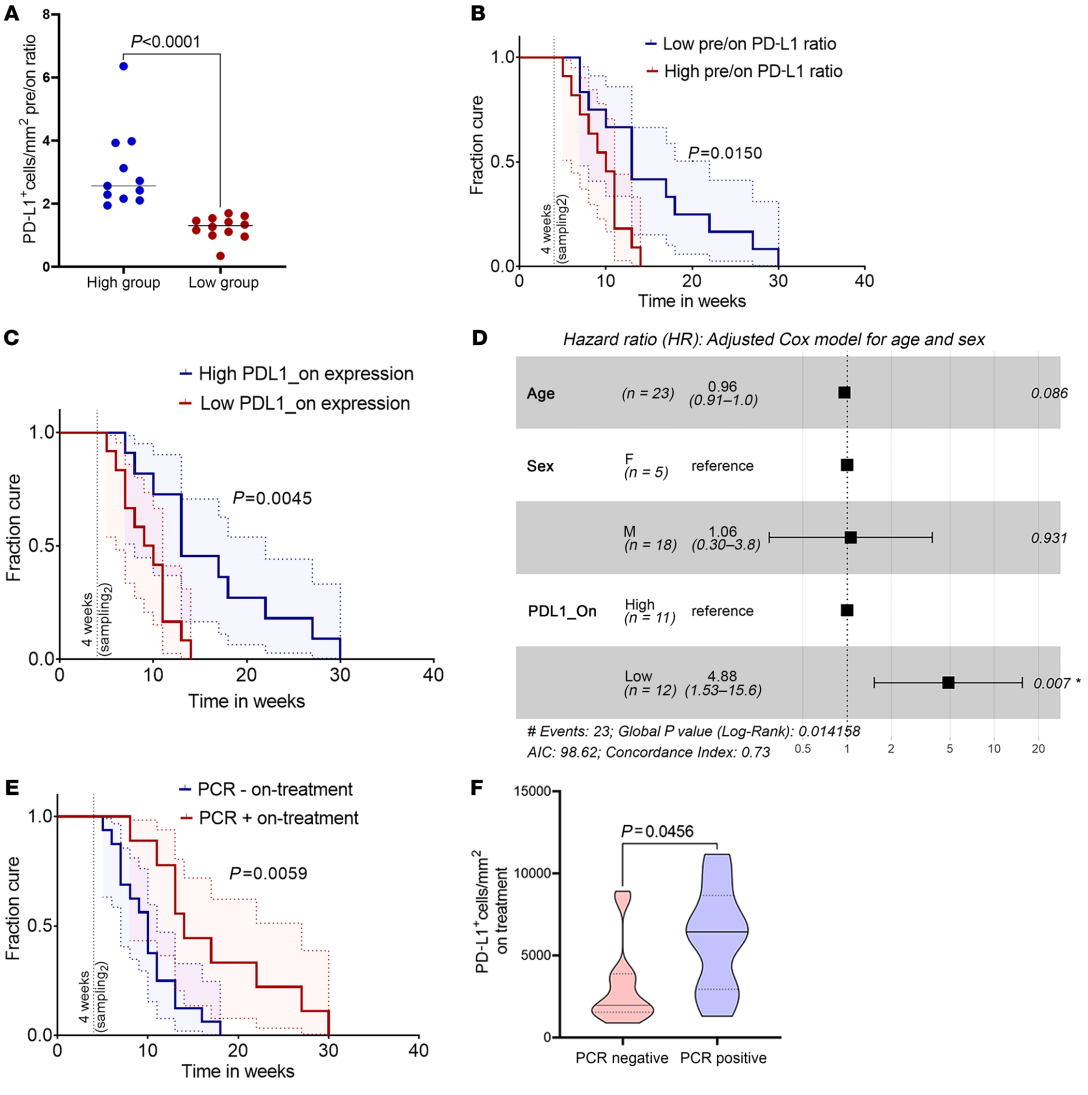
Clinical correlates of PD-L1 reduction on treatment in patients with CL. (**A**) Patients (validation cohort; *n =* 23) were stratified based on high (> geomean value; *n =* 11) and low (< geomean value; *n =* 12) pre-/on-treatment expression ratio. (**B**) Kaplan-Meier curve based on pre-/on-treatment ratio of PD-L1 expression (high versus low). (**C**) Patients stratified based on on-treatment expression of PD-L1 (> geomean value; *n =* 11 versus < geomean value; *n =* 12). (**D**) Multivariate Cox proportional hazards model plotted as a forest plot. *P* values for each covariate represent Wald statistic value, and overall statistical significance is also indicated. (**E**) Patients stratified by *LITS1* PCR status (*n =* 9 PCR^+^ versus *n =* 14 PCR^–^ or PCR^+/–^ [equivocal]) on treatment. (**F**) PD-L1 expression in *LITS* PCR^+^ versus PCR^–^ individuals on treatment. Dotted lines show upper and lower quantile, solid line shows median. *P* value generated using 2-tailed Mann-Whitney test. Vertical line drawn in **B**, **C**, and **E** on the *x* axis shows time when on-treatment biopsies were collected. Curves in **B**, **C**, and **E** were compared using log-rank (Mantel-Cox) test. Blue and red shaded areas show 95% CI of the 2 groups.

Finally, we tested whether reduction in IDO1 or PD-L1 expression early during therapy could be used as a prognostic marker for treatment response. Patients with the greatest reduction in PD-L1 expression (i.e., greater than the geomean of the pretreatment: on-treatment expression ratio; *n =* 12 patients; Figure 4, A and B) were cured earlier than those who had lower or no reduction in PD-L1 expression (*P =* 0.015). Patients with lower PD-L1 expression after 4 weeks of treatment (i.e., lower than the geomean of on-treatment expression; *n =* 12 patients) also were cured faster (*P =* 0.0045; Figure 4C). We assessed the association of PD-L1 with disease cure rate using univariate Cox proportional hazard regression (Supplemental Figure 13A; HR = 3.96, *P =* 0.008). Upon adjustment for age and sex of the participants, HR increased to 4.88 (*P =* 0.007; Figure 4D), indicating that patients with maximally reduced PD-L1 expression upon treatment were about 5 times more likely to be cured earlier. Conversely, patients remaining parasite PCR^+^ at 4 weeks after treatment had a significantly longer cure time (Figure 4E) and higher PD-L1 expression (Figure 4F). Surprisingly, reduction in IDO1 expression, calculated as either pretreatment/on-treatment expression ratio or IDO1 expression at 4 weeks (*n =* 12 versus *n =* 11), did not correlate with cure rate (Supplemental Figure 13, B and C). Thus, the relationship between declining PD-L1 expression and rate of cure appears selective.

We conclude that expression of IDO1 and PD-L1 immune checkpoint molecules is a common feature of Sri Lankan CL and that intracellular parasitism is associated with heightened expression of these immunoregulatory proteins in lesional macrophages. Tissue expression of both IDO1 and PD-L1 is markedly reduced within 2 to 4 weeks of treatment onset and well in advance of clinical cure, and a reduction in PD-L1 is associated with a more rapid therapeutic response. The elevated expression of negative immune regulators on macrophages at the lesion site, as demonstrated here, has clear parallels with tumor-associated macrophages (36) and extends our understanding of how *Leishmania* parasites influence the function of their host cell during human disease (37). Though longitudinal sampling of the same macrophage population was not possible, it seems likely that reduction of PD-L1 expression is facilitated by the leishmanicidal action of SSG, suggesting a model for drug-immune synergy whereby early rounds of SSG treatment reduce intracellular parasite burden, leading to reduced checkpoint inhibition and reengagement of T cell effector function. Our data, together with strong preclinical evidence of an inhibitory role of PD-L1 in various forms of leishmaniasis (10, 12, 38), support the candidacy of PD-L1 blockade as an adjunct HDT in Sri Lankan CL. In addition, our data suggest the possibility that changes in PD-L1 expression early after treatment could be considered as a biomarker to trigger drug tapering or drug cessation.

## Methods

A complete, detailed description of Methods is provided in the supplemental material.

### Study approval.

The study was conducted in accordance with the principles of the Declaration of Helsinki and was approved by the Ethical Review Committee of the Faculty of Medical Sciences, University of Jayewardenepura (ref: 780/13 & 52/17) and the Department of Biology, University of York. Written informed consent, including for lesion photographs, was received from participants prior to inclusion in this study.

## Author contributions

NSD, SS, VS, NPM, BS, MMDO, LCR, SM, and SR conducted experiments. NSD and SF performed data analysis. NSD and PMK wrote the manuscript. PBW, MC, HG, RW, DL, PMK, and SR were involved in conceptualization and securing funding. PMK and SR supervised the study. The order of the co–first authors was determined by their relative contributions to the study.

## Supplementary Material

Supplemental data

## Version 1 10/05/2021

In-Press Preview

## Version 2 11/15/2021

Electronic publication

## Version 3 12/13/2021

License information corrected (CC BY 4.0)

## Version 4 03/01/2024

Correction to figure 2
